# Water-Mediated Stability of Ionic Liquids Confined in Aqueous Droplets: Molecular Dynamics Insights with Atmospheric Parallels

**DOI:** 10.1021/acs.jpcb.5c06039

**Published:** 2025-11-06

**Authors:** Tertius Lima Fonseca, Guilherme Colherinhas

**Affiliations:** Instituto de Física, 67824 Universidade Federal de Goiás, 74690-900 Goiânia, GO Brazil

## Abstract

This study investigates the structural and volumetric effects of protic ([cho]­[gly]) and aprotic ([emim]­[BF_4_]) ionic liquids (ILs) on confined aqueous bubbles via molecular dynamics simulations. Pure water bubbles exhibit near-spherical symmetry and slight volumetric expansion (∼4%) with increasing temperature, consistent with thermal expansion. The introduction of ILs induces significantly larger volume increases (up to 36% at high ion concentrations) due to ionic insertion at bubble interfaces, which reorganizes the hydrogen bond (HB) network and expands the structure. Protic ILs show stronger stabilizing effects than aprotic ILs, attributed to cooperative HB formation with water and among ions. Dynamic analyses reveal that ILs maintain or increase HB numbers and lifetimes while raising rupture energy barriers, reinforcing bubble cohesion and thermal resilience. Despite volumetric changes, bubble morphology and water density symmetry remain intact, highlighting ILs as active structural stabilizers. Beyond nanoconfined IL–water systems, these water-mediated stabilization mechanisms parallel atmospheric processes in which hygroscopic growth, cloud condensation nuclei (CCN) activation, and particle lifetimes are governed by interfacial organization. The molecular descriptors established here (HB lifetimes, rupture free-energy (Δ*G*), Coulombic/Lennard–Jones interaction energy (*E*_C_/*E*_LJ_) trends, density symmetry) provide transferable parameters for more realistic representations of water uptake and activation in environmental models.

## Graphical abstract

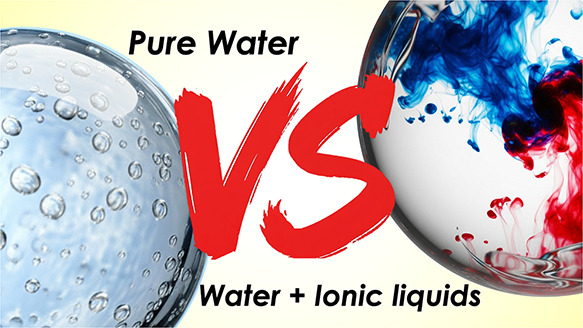

## 1 Introduction

Water is extensively studied through molecular dynamics (MD) simulations, particularly under periodic boundary conditions that mimic an isotropic and macroscopic system. In such setups (typically conducted in the NPT ensemble) the system is modeled as infinite, with the simulation box completely filled with water molecules. Under these conditions, the influence of temperature can be monitored through variations in volumetric properties, such as density and pressure. However, these simulations do not allow for the observation of global structural effects, such as morphological deformations or local instabilities, since there are no free interfaces, geometric anisotropies, or surfaces that would allow for spontaneous cohesive rupture. To investigate the structural response of water in confined environments or with free boundaries, an alternative approach consists of simulating a finite portion of water isolated in a sufficiently large box, such that interactions with its own periodic images are minimized. A particularly interesting configuration is that of a spherical water droplet immersed in computational vacuum, which can be used as a model to study the stability of the liquid phase under extreme conditions, such as those involved in evaporation, cavitation, or gas-phase nucleation.^1,2^ Although no solid boundaries are present, this configuration represents a finite and spatially confined system, where molecular motion is restricted by the liquid–vacuum interface. Such “free-boundary confinement” has been extensively employed in molecular dynamics studies of nanodroplets and nanobubbles,^1,2^ as it captures the structural and dynamic effects arising from reduced coordination and finite-size limitations relative to bulk water. In this scenario, temperature variation allows for the evaluation of how the system responds to thermal fluctuations, especially in terms of molecular cohesion and the stability of the liquid–vapor interface.

As the temperature increases, the gain in kinetic energy by surface molecules promotes the disruption of hydrogen bonds and, consequently, the potential evaporation of molecules from the droplet surface. This phenomenon can be monitored in MD by tracking the number of molecules that move away from the droplet’s center of mass, as well as by analyzing local potential energy and intermolecular interactions.^2^ Droplet stability is therefore directly linked to the robustness of the hydrogen bond network, whose average number and lifetime tend to decrease with increasing temperature.^3,4^ In this context, a relevant scientific question is to understand which factors can delay or inhibit the disintegration of the liquid droplet at elevated temperatures. It is well-known that the addition of solutes, such as salts or ionic liquids (ILs), can significantly alter the thermodynamic and structural properties of water.^5,6^ In particular, protic ionic liquids (PILs)—whose cations can donate hydrogen bonds (HBs)—have the potential to strengthen water–water cohesion by forming additional HBs and increasing the local density of directional interactions.^5,7^ On the other hand, nonprotic ionic liquids (N-PILs), although they also interact with water, do so mainly through long-range electrostatic interactions and dispersion forces. These interactions, while important, are less specific and less cooperative than hydrogen bonding. Therefore, a direct comparison between the influence of PILs and N-PILs on droplet stability can reveal the extent to which hydrogen bonding is crucial for maintaining system cohesion under thermal stress. Additionally, the effects of different additives on droplet stability can be quantified using well-established MD tools, such as the analysis of the number and lifetime of hydrogen bonds.^8,9^ These parameters allow for the evaluation of how ionic liquids stabilize the droplet interface and offer insights into molecular mechanisms relevant to natural and technological processes involving water phase transitions.

Recent studies have explored how the presence of ionic liquids (ILs) modulates the behavior of water under various thermodynamic and interfacial conditions. For instance, it has been demonstrated that water molecules can be retained or selectively removed from ionic liquid domains depending on local microstructures, influencing the hydrogen bond network and overall system organization.^8^ Additionally, spectroscopic and molecular simulations have shown that protic ILs (PILs), such as those containing imidazolium-based cations, form stronger and more directional hydrogen bonds with water compared to aprotic ILs (N-PILs), stabilizing hydration shells and promoting aggregation at low concentrations.^5,6^ This bonding capacity is especially evident in PILs with highly basic anions like [Cl]^−^ or [HSO_4_]^−^, which interact strongly with water and hinder molecular evaporation under thermal perturbation.^5^

The ability of ILs to modulate evaporation and cohesion is not only limited to chemical composition but also extends to thermoresponsive behavior. IL/water mixtures exhibiting lower critical solution temperature (LCST) behavior can transition from fully miscible to phase-separated states under moderate heating, with the water–IL interaction pattern shifting drastically near the transition point.^10,11^ These reversible transitions have been leveraged in catalytic systems to isolate products and recycle ILs simply by adjusting the temperature.^11^ From a structural standpoint, molecular dynamics simulations have shown that even small concentrations of ILs can enhance the hydrogen-bonding network within water clusters and droplets, effectively delaying interfacial instability and molecular ejection.^7^ The solubility trends and interaction strengths depend not only on the nature of the anion but also on the hydrogen-bonding capabilities of the cation, as confirmed by both QSPR models and MD data.^7^

Furthermore, MD studies involving droplet evaporation, condensation, and cavitation on solid surfaces reveal that external conditions—such as surface energy, geometry, and electric fields—play a crucial role in droplet cohesion and stability.^1,3,4,9,12,13^ For example, when water droplets are subjected to electric fields, dipolar alignment induces anisotropic deformation, which can either promote or suppress evaporation depending on field strength and direction.^4^ Similarly, studies of droplet dynamics on hydrophilic and hydrophobic nanostructured surfaces have shown that the integrity of the hydrogen bond network determines the onset of vapor nucleation or droplet collapse.^1,3^ These findings underscore that both intrinsic molecular interactions and external perturbations govern the resilience of water droplets—highlighting the relevance of exploring additive effects, such as those induced by PILs and N-PILs, under mild thermal conditions as pursued in the present study.

A complementary perspective on droplet stability emerges from studies that focus exclusively on the intrinsic molecular structure of nanodroplets, even in the absence of solutes. Recent molecular dynamics simulations have demonstrated that the structural integrity of water droplets is not uniform throughout their volume: while the core retains a well-defined hydrogen-bond network, the interfacial region exhibits significant disruption, marked by reduced density and increased molecular disorder.^14^ These effects are amplified as droplet size decreases or temperature increases, revealing a natural predisposition toward interfacial destabilization. Such findings underscore the importance of localized structural metrics—like tetrahedrality and radial coordination distances—as critical indicators of cohesion, and further justify the investigation of external agents, such as ionic liquids, that may help preserve interfacial order under thermal stress.

In this work, we conduct a molecular dynamics study of a spherical water droplet composed of 2000 water molecules, inserted in a cubic box large enough to eliminate periodic artifacts. Temperature is systematically varied to induce interface destabilization. Three systems are compared: (i) a pure water droplet, (ii) a droplet containing a fraction of a protic ionic liquid ([cho]­[gly]), and (iii) a droplet containing a nonprotic ionic liquid ([emim]­[BF_4_]). The main objective is to investigate how different types of intermolecular interactions (especially hydrogen bonding promoted by PILs) affect the thermal stability of the droplet and delay or prevent water evaporation. The ionic liquids [cho]­[gly] and [emim]­[BF_4_] represent two chemically and structurally distinct classes of ionic media that interact differently with water, influencing hydrogen-bond networks and interfacial cohesion. The protic [cho]­[gly] belongs to the family of cholinium amino acid ionic liquids, characterized by strong hydrogen-bonding capabilities between the hydroxyl group of the cholinium cation and the carboxylate moiety of the glycinate anion. Molecular dynamics studies have demonstrated that [cho]­[gly] exhibits extensive intra- and interionic hydrogen-bond networks, including antiparallel arrangements between like-charged cations stabilized by O–H···O and C–H···O interactions.^15,16^ These features confer high cohesion and biocompatibility, making [Cho]­[Gly] a representative bioionic liquid with reduced volatility and strong hydrophilic character.^17^ In contrast, [emim]­[BF_4_], an aprotic room-temperature ionic liquid, presents dominant electrostatic and dispersion interactions.^18,19^ Its anion, tetrafluoroborate (BF_4_^–^), favors weak solvation of water and reduced association constants in high-permittivity environments, resulting in higher mobility and lower viscosity compared to [cho]­[gly]. Diffusion studies based on free volume theory indicate coordinated motion of cation–anion pairs and enhanced translational dynamics at elevated temperatures.^20^ Therefore, [cho]­[gly] is expected to stabilize the water droplet primarily through cooperative hydrogen-bond reinforcement, while [emim]­[BF_4_] should promote mobility and structural rearrangement via electrostatic screening. The contrast between these two interaction regimes provides a molecular framework to evaluate how hydrogen bonding versus purely ionic coupling modulates droplet integrity under thermal stress.^21^ Through the analysis of structural, dynamic, and thermodynamic properties, we aim to establish structure–property relationships under small heating (*T* = 300 K and *T* = 323 K).

The behavior observed in IL–water nanoconfinement finds direct parallels in atmospheric particle physics. Water acts as a universal mediator, regulating hygroscopicity and cloud condensation nuclei (CCN) activation, as formalized by the κ-parameter framework of Petters and Kreidenweis.^22^ Beyond this, water actively organizes mixed phases, preventing phase separation and sustaining morphological integrity, consistent with the evolution of secondary organic aerosols (SOA) discussed by Kroll and Seinfeld^23^ and Jimenez et al.^24^ Chemical composition further modulates interfacial stability: protic ILs behave analogously to hydrophilic atmospheric organics, while aprotic ILs resemble less polar constituents, echoing the role of functional chemistry in water uptake and reactivity highlighted by Ziemann and Atkinson.^25^ Finally, the ability of water and interfacial organization to preserve cohesion under stress resonates with observations of amorphous SOA states, where restricted diffusion and strong interfacial bonding dictate atmospheric particle persistence and activation (Virtanen et al.,^26^).

## 2 Methodology

In this study, classical molecular dynamics (MD) simulations were performed to investigate the thermal stability of spherical droplets of water and water–ionic liquid (IL) mixtures confined within a vacuum region inside a large simulation box, avoiding self-interaction of the water droplet with its replicas due to periodic boundary conditions. Three systems were analyzed: pure water (H_2_O), water with protic ionic liquid ([cho]­[gly]), and water with nonprotic ionic liquid ([emim]­[BF_4_]). The simulations were conducted using the Gromacs package,^27,28^ with conditions suitable for representing nonperiodic systems containing liquid–vacuum interfaces. The ionic liquids chosen for this study are commonly used in studies of traditional and biodegradable supercapacitors.^29–33^

The simulated droplets were composed of 2000 water molecules modeled using the TIP3P force field,^34,35^ initially arranged in a geometry approximately spherical, with a density consistent with liquid water at room temperature. For the systems containing ionic liquids, 20 or 40 ionic pairs of each compound were added. The choline cation and the glycine anion ([cho]­[gly]) were chosen as the model for the protic ionic liquid due to the presence of functional groups capable of forming HBs with the water molecules in the droplet. The force field used for this ionic pair was.^36^ The pair composed of 1-Ethyl-3-methylimidazolium tetrafluoroborate ([emim]­[BF_4_]) was used as an example of a nonprotic ionic liquid, with interactions dominated by electrostatic and dispersion forces. The force field used for the nonprotic ionic pair was based in.^37^ All systems were inserted into cubic boxes approximately 7 × 7 × 7 nm^3^, sufficiently large to avoid artificial interactions between the periodic images of the droplet. All systems were constructed by randomly distributing the molecules/ions in the spherical region using the Packmol program,^38^ as illustrated in Figure 1.

**Figure 1:**
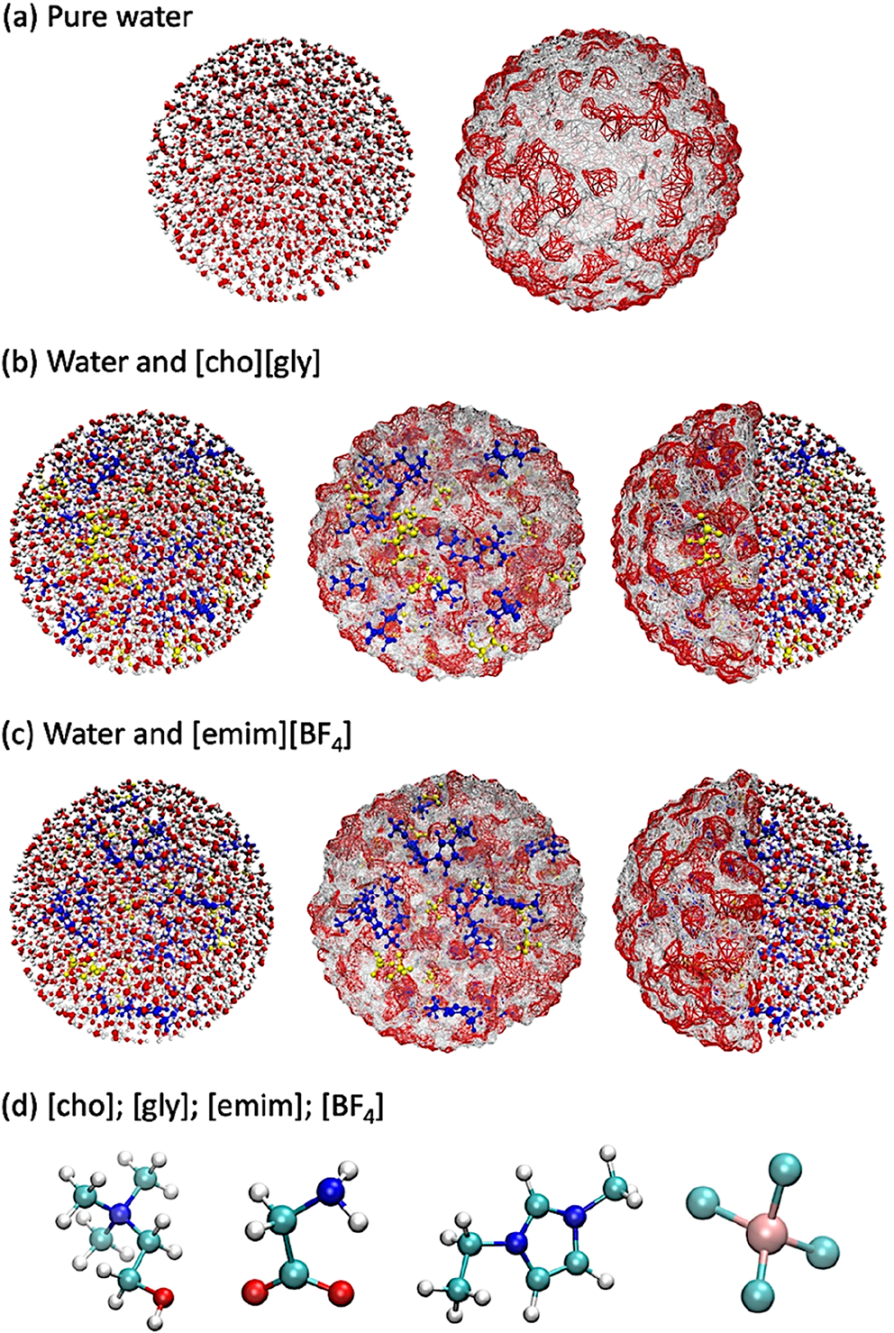
Initial configurations of the simulated water bubbles. (a) Pure water, highlighting molecular detail and surface structure. (b) Water containing the protic ionic liquid [cho]­[gly] (40 ion pairs), with [cho] shown in blue and [gly] in yellow. (c) Water containing the aprotic ionic liquid [emim]­[BF_4_] (40 ion pairs), with [emim] in blue and [BF_4_] in yellow. (d) Molecular structures of [cho], [gly], [emim], and [BF_4_] ions. Atom color scheme: O (red), N (blue), F (pink), H (white), C (dark cyan), and B (light cyan).

The simulations were carried out in the NVT ensemble, with the temperature kept constant through a v-rescale thermostat^39^ with a coupling constant of 0.1 ps. The simulated temperatures were 300 and 323 K, allowing for the analysis of the thermal evolution of the systems on conditions close to room temperature. van der Waals and Coulomb interactions were treated with a cutoff radius of 1.2 nm and the PME (Particle Mesh Ewald) method^40^ for the long-range electrostatic interactions. Periodic boundary conditions were maintained only for computational integrity purposes, although the distance between the droplet and the box walls was adjusted to minimize any periodic effects. Equilibration and production simulations were performed with a time step of 1 fs. A total of 50 ns was used for the thermodynamic equilibration period of the droplet in the simulation box, while 100 ns of simulation time was used for the production step for each system, with data collected every 100 fs for subsequent analysis. The LINCS algorithm^41^ was used to maintain the integrity of the molecules and thus of the classical simulation. For analysis, the water droplet was recentered in the simulation box, ensuring that the center of mass served as a reference for the comparisons to be made. Visualizations and initial structural analyses were carried out with the aid of the VMD program^42^ and GROMACS software packages.^27,28^ The Packmol program^38^ was used to model the initial configurations. This methodology is well accepted to describe hydrated ionic liquids according to references.^31,33,43–45^ Additionally, the translational dynamics of water molecules and ionic species were characterized through mean square displacement (MSD) analyses and diffusion coefficients ( D ) obtained from the Einstein relation, as implemented in the GROMACS suite.^27,28^ MSD curves were computed for the center of mass of water molecules and for each ionic species ([cho], [gly], [emim], [BF_4_]), considering the full droplet ensemble. The diffusion coefficient was extracted from the linear regime of ⟨*r* ^2^(*t*)⟩ versus time, within the 10–20 ns window of the production trajectory for all systems. Due to the strong positional exchange of molecules across the droplet, a spatial decomposition into “core” and “interfacial” regions was deemed physically inconsistent and was therefore not applied. The results thus represent average translational mobility within the entire confined system, which remains meaningful for assessing thermal and ionic effects on molecular dynamics in nanoconfined droplets.

The analyses included: (i) variations of average interaction energy (Lennard-Jones + Coulomb) of water–water interactions as a function of temperature and composition of ionic liquids, (ii) monitoring of the droplet’s structural cohesion, and (iii) average number and lifetime of hydrogen bonds of the water–water interactions. These metrics allowed for the evaluation of the role of specific interactions (especially hydrogen bonds in the case of PILs) in preserving the structural integrity of the droplet under *T* = 300 K and *T* = 323 K. Figure 1 highlights the simulated systems, and Table 1 outlines the composition of each system.

**Table 1:** Composition of the Simulation Boxes Studied in This Work

	pure water	water-[cho][gly]	water-[emim][BF_4_]
# water molecules	2000	2000	2000
#ionic Liquids pair	--	System-A1 = 20	System-A2 = 20
		System-B1 = 40	System-B2 = 40

## 3 Results and Analysis

### 3.1 Energy Interactions

The total interaction energy between water molecules (*E*_Total_ = *E*_Coulomb_ + *E*_Lennard–Jones_) was evaluated to understand the effects of introducing the ionic liquid [cho]­[gly] into a confined water bubble. Our results indicate a progressive decrease in the average *E*_Total_ as the concentration of ionic pairs increases: approximately 3.6% for System-A1 (20 pairs), and 6.7% for System-B1 (40 pairs). This decline suggests a weakening of the water–water hydrogen-bonding network, reducing the overall cohesiveness of the system. Notably, this trend appears to be largely independent of temperature, as thermal variations had only a marginal influence on these values. The interaction energy between [cho] cations and water molecules also exhibits a decreasing trend with increasing ionic concentration. Comparing Systems-A1 and B1 reveals an average reduction of ∼8%. Once again, temperature (*T* = 300 K nd *T* = 323 K) plays a minor role, indicating that these energetic changes are predominantly driven by the structural reorganization of the system rather than thermal agitation. In the case of interactions between [gly] anions and water molecules, a smaller but still noticeable decrease in total interaction energy is observed with increasing ion concentration. This behavior is expected due to the potential for hydrogen bonding between the [gly] anion and water, which provides a stabilizing effect. The comparison (Systems-A1 vs B1) show an average decrease of around 5%, suggesting a nearly linear trend as ionic content increases. Importantly, when comparing the interaction energy of water with [cho] or [gly] to that of water–water within the same system, it becomes evident that ion–water interactions are significantly stronger. For example, in System-A1, the water-[cho] and water-[gly] interactions are approximately 223% and 519% (224% and 529%) greater than water–water interactions, respectively. These values slightly decrease with increasing ion concentration, reaching ∼211% and ∼509% (213% and 517%) in System-B1, respectively, for *T* = 300 K (*T* = 323 K). This emphasizes the dominant role of ion–water interactions in maintaining system cohesion despite the weakening water–water network. These trends are illustrated in Figure 2a.

**Figure 2:**
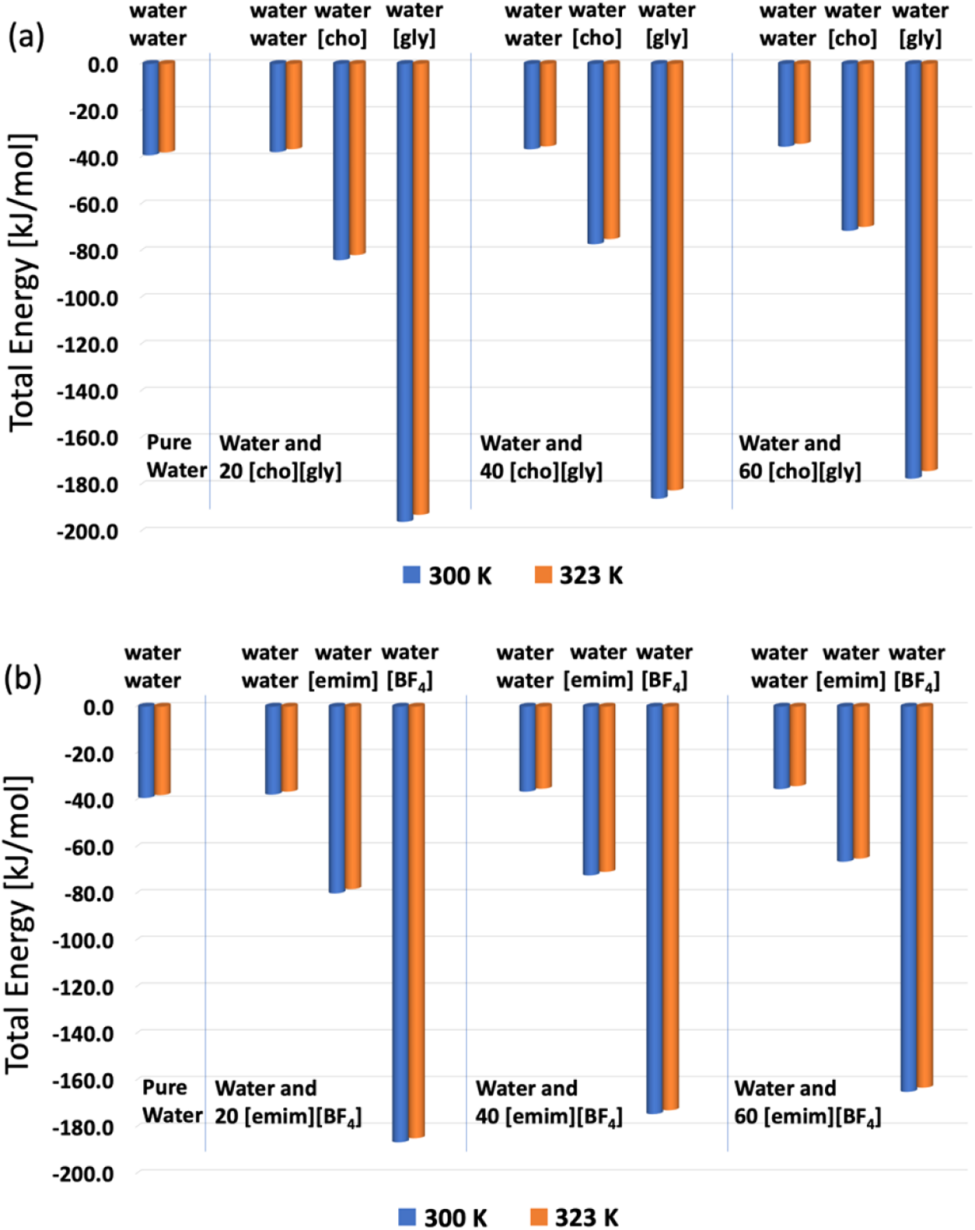
Total interaction energy (Coulomb + Lennard–Jones) for the studied systems. The analyzed interactions include: water–water (per water molecule in solution) and water–ion (per ionic unit in solution). Energies are expressed in kJ/mol and correspond to all simulated ionic concentrations and simulated temperatures. (a) System composed of water and the protic ionic liquid [cho]­[gly]. (b) System composed of water and the aprotic ionic liquid [emim]­[BF_4_].

Figure 2b presents the corresponding results for systems containing the aprotic ionic liquid [emim]­[BF_4_] (to make the replacement [cho]­[gly] from [emim]­[BF_4_]). The water–water interaction energies in these systems follow the same decreasing trend observed previously. However, the magnitude of the ion–water interactions is somewhat lower than in the [cho]­[gly] systems. Specifically, water-[emim] and water-[BF_4_] interactions are ∼212% and ∼496% (215% and 508%) stronger than water–water interactions in System-A2, and decreasing to ∼199% and ∼479% (201% and 492%) in System-B2, for *T* = 300 K (*T* = 323 K). The total water–ion interaction energy also decreases by ∼8% from System-A2 to B2, reflecting reduced water structuring around the aprotic ionic liquid components. These results support the hypothesis that aprotic ionic liquids such as [emim]­[BF_4_] induce less organization of water molecules in their vicinity compared to protic systems, leading to a more disrupted and less cohesive water bubble. Nevertheless, the ion–water interaction energies remain high (primarily due to strong electrostatic contributions) ensuring the structural integrity of the bubble + ion system. In particular, water-[gly] and water-[BF_4_] interactions are especially relevant, with energies ranging from −195 to −172 kJ/mol per ion in solution. Finally, across all systems and interaction types, a slight but consistent decrease in interaction energy was observed with increasing simulated temperature. This reduction follows a nearly linear trend, as shown in Figure 2, and reflects the expected thermal weakening of noncovalent interactions in molecular systems.

The analysis of average Coulomb interaction energy (*E*_C_ per-pair), for Systems-A1 e B1, shows that increasing the concentration of ionic liquids (from 20 to 40 pairs of [cho]­[gly]) results in a weakening of electrostatic interactions between water and the ions. For the water–[cho] pair, the E_C_ energy decreases from −42.6 kJ/mol to −38.4 kJ/mol at 300 K, representing a reduction of approximately 10%. In the case of water–[gly], the decrease is from −187.8 kJ/mol to −179.0 kJ/mol, corresponding to a variation of about 5%. Upon increasing the temperature to 323 K, a further decrease in *E*_C_ is observed: around 0.2% for water–[cho] and 2.2% for water–[gly]. These results suggest that the higher ionic concentration alters the system’s environment due to increased competition for solvation sites and reorganization of the water hydrogen-bond network. Thermal elevation further contributes to the weakening of these interactions by increasing molecular mobility and thermal agitation.

The van der Waals Interactions energy (*E*_LJ_, per-pair) is also significantly affected by the increase in ionic pair concentration. The average *E*_LJ_ per pair for the water–[cho] system decreases from −41.3 kJ/mol to −38.7 kJ/mol at 300 K (a reduction of approximately 6%), while for water–[gly], the decrease is from −8.1 kJ/mol to −7.0 kJ/mol, corresponding to ∼14%. Increasing the temperature to 323 K further weakens *E*_LJ_ for water–[cho], with values decreasing from −38.7 to −36.6 kJ/mol (−5.4%). Interestingly, for water–[gly], *E*_LJ_ increases slightly from −7.0 to −7.4 kJ/mol (+5.7%), suggesting a possible local structural rearrangement that enhances van der Waals interactions with rising temperature. Overall, these results indicate that *E*_LJ_ is sensitive to both ionic density and temperature, reflecting changes in the compactness and structural organization of the system under different thermodynamic conditions.

For the electrostatic interaction energy (*E*_C_) for System-A2 e B2, increasing the number of [emim]­[BF_4_] ion pairs from 20 to 40 leads to a noticeable decrease in the average water–[emim] interaction per ion pair: from −35.0 kJ/mol to −29.8 kJ/mol at 300 K, and from −35.2 kJ/mol to −30.2 kJ/mol at 323 K. This corresponds to a relative weakening of about 15% at 300 K and 14% at 323 K. A similar trend is observed for the water–[BF_4_] interaction, where E_C_ drops from −184.7 kJ/mol to −173.4 kJ/mol at 300 K, and from −182.7 kJ/mol to −171.5 kJ/mol at 323 K (∼6% reduction). These results indicate that electrostatic interactions between water and both ions become weaker with higher ion concentration, possibly due to increased competition among ions for hydration sites or greater ionic screening. Regarding the Lennard-Jones interaction energy (*E*_LJ_), a similar dilution effect is observed. For water–[emim], the interaction decreases from −44.9 kJ/mol to −42.5 kJ/mol at 300 K (∼5% reduction), and from −42.9 kJ/mol to −40.5 kJ/mol at 323 K (∼6% reduction). For water–[BF_4_], the change is even more pronounced: *E*_LJ_ shifts from −1.8 kJ/mol to −1.0 kJ/mol at 300 K (∼44% reduction) and from −2.1 kJ/mol to −1.3 kJ/mol at 323 K (∼38% reduction). These findings suggest that the van der Waals component of the interaction becomes considerably weaker as ion concentration increases, especially for the water–[BF_4_] pairs, reinforcing the idea of reduced structuring and cohesion in the hydration shell at higher ion densities. Table 2 summarizes the electrostatic (*E*_C_) and van der Waals (*E*_LJ_) interaction energies obtained for all systems and temperatures.

**Table 2:** Average Electrostatic (*E*_C_), Lennard–Jones (*E*_LJ_), and Total Interaction Energies Obtained for the Water Droplet in the Absence and Presence of Ionic Liquids at 300 and 323 K (Per Number of Waters in Water–Water Interaction or Number of Ions in Ion-Water Interaction)^a^

system	*T* (K)	*E*_C_ (kJ/mol)	*E*_LJ_ (kJ/mol)	total (kJ/mol)
Pure Water → water–water	300	–43.7	4.6	–39.1
Pure Water → water–water	323	–42.2	4.3	–37.9
System-A1 → water–water	300	–42.4	4.7	–37.7
System-A1 → water–water	323	–40.9	4.4	–36.5
System-A1 → [cho]-water	300	–42.6	–41.3	–83.9
System-A1 → [cho]-water	323	–42.6	–39.2	–81.8
System-A1 → [gly]-water	300	–187.8	–8.1	–195.9
System-A1 → [gly]-water	323	–184.2	–8.7	–192.9
System-B1 → water–water	300	–41.3	4.8	–36.5
System-B1 → water–water	323	–39.7	4.4	–35.3
System-B1 → [cho]-water	300	–38.4	–38.7	–77.1
System-B1 → [cho]-water	323	–38.3	–36.6	–74.9
System-B1 → [gly]-water	300	–179.0	–7.0	–186
System-B1 → [gly]-water	323	–175.0	–7.4	–182.4
System-A2 → water–water	300	–42.3	4.7	–37.6
System-A2 → water–water	323	–40.8	4.4	–36.4
System-A2 → [emim]-water	300	–35.0	–44.9	–79.9
System-A2 → [emim]-water	323	–35.2	–42.9	–78.1
System-A2 → [BF_4_]-water	300	–184.7	–1.8	–186.5
System-A2 → [BF_4_]-water	323	–182.7	–2.1	–184.8
System-B2 → water–water	300	–41.1	4.7	–36.4
System-B2 → water–water	323	–39.5	4.4	–35.1
System-B2 → [emim]-water	300	–29.8	–42.5	–72.3
System-B2 → [emim]-water	323	–30.2	–40.5	–70.7
System-B2 → [BF_4_]-water	300	–173.4	–1.0	–174.4
System-B2 → [BF_4_]-water	323	–171.5	–1.3	–172.8

a All values were computed from equilibrium molecular dynamics trajectories using time-averaged nonbonded energy components. Systems A1 and B1 correspond to [cho]­[gly]-water droplets, while systems A2 and B2 correspond to [emim]­[BF_4_]-water droplets.

The comparison between the water–[cho]­[gly] and water–[emim]­[BF_4_] systems reveals significant differences in the strength of ion–water interactions, which can be attributed to the distinct nature of the ionic liquids used. In the system with the protic ionic liquid [cho]­[gly], stronger ion–water interactions are observed, both electrostatic and dispersive (*E*_C_ and *E*_LJ_), especially with the [gly]^−^ anion, whose hydroxyl functional groups promote stable hydrogen bonding with water molecules. In contrast, the system containing the aprotic ionic liquid [emim]­[BF_4_] exhibits significantly weaker ion–water interactions, suggesting a lower ability to organize water molecules around the ions, particularly due to the lower polarizability of the imidazolium cation and the limited hydrogen bonding capability of [BF_4_]^−^. This structural and functional difference between the ionic liquids directly impacts the cohesion of the aqueous bubble and the stability of the noncovalent interaction network, making the system with [cho]­[gly] more prone to forming organized and stable structures in aqueous solution.

### 3.2 Mass Density Profile

Although a radial density profile would be the most natural choice for a perfectly spherical droplet, the inclusion of ionic liquids introduces subtle anisotropic effects that can locally distort the droplet shape. To capture these possible deviations from spherical symmetry, the number density was projected along the three Cartesian axes (*x*, *y*, and *z*). This approach enables direct comparison of the droplet dimensions along independent directions, serving as a more sensitive descriptor of potential shape anisotropies induced by ionic structuring or thermal fluctuations. Figure 3 shows the number density profile of the system (for water molecules) projected along the axes of the simulation box. For this analysis, all configurations from the classical trajectory obtained via molecular dynamics under thermodynamic equilibrium were centered. As can be seen, for the structure containing only water molecules (Figure 3a), the density profile displays a nearly spherical shape with a symmetric distribution, where the peak corresponds to the center of the water bubble. An analysis of the full width at half-maximum (FWHM) of the mass density profile provides a good estimate of the average diameter of the structure and its projection along the three Cartesian axes.

**Figure 3:**
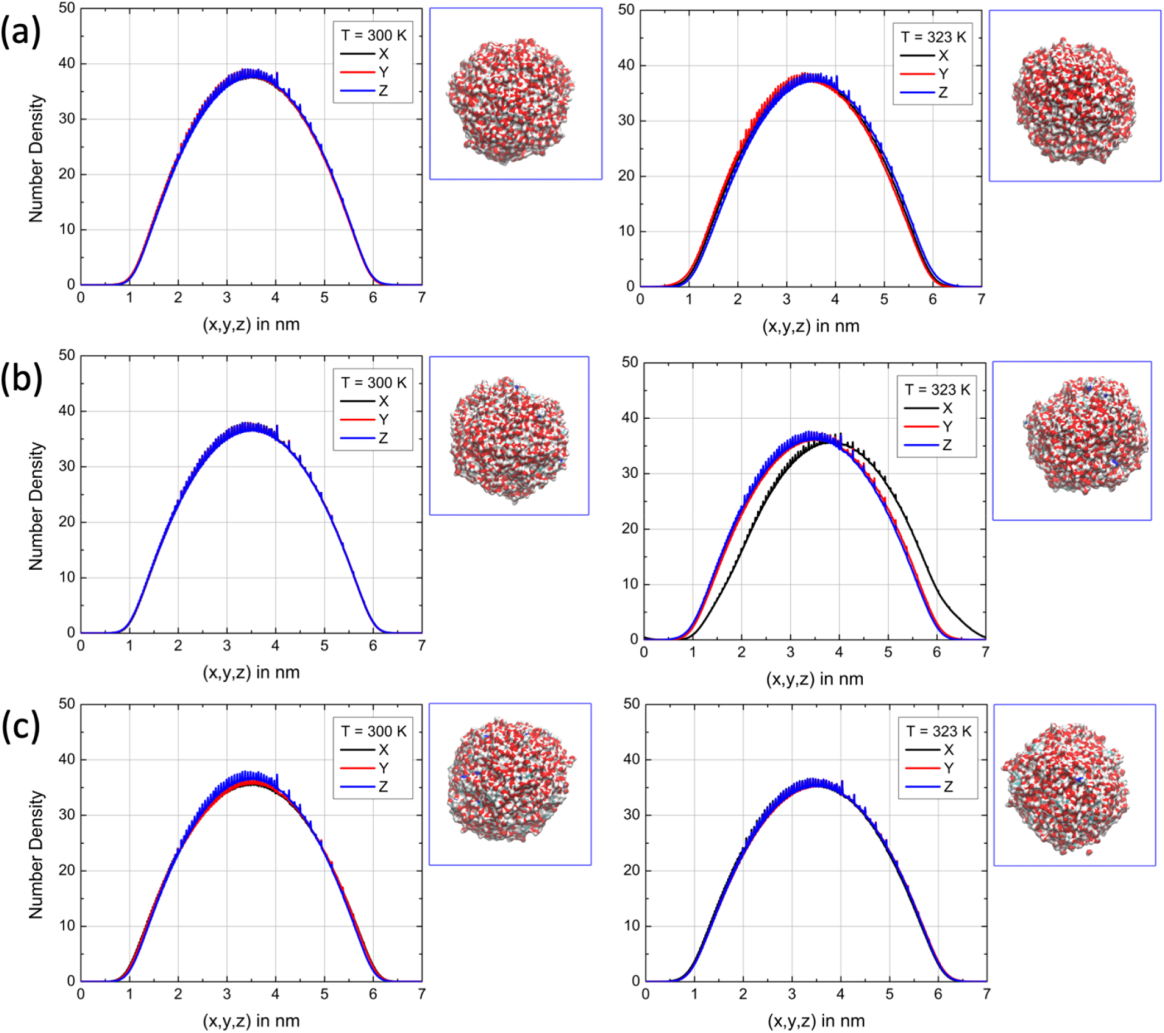
Final configuration from the simulation of (a) the pure water bubble; water bubble with (b) 20 pairs of [cho]­[gly]; and (c) 40 pairs of [cho]­[gly]. The number density projections along the three axes of the system are also shown for a simulation box with fixed edges of 7 nm. The *XY* plane is highlighted to illustrate the shape of the bubble. At 323 K, a slight displacement of the *x*-axis density profile can be observed, which arises from transient thermal fluctuations and minor interfacial reorganization. This effect is not systematic and remains within the expected variability of the droplet’s quasi-spherical structure.

The results show that the average bubble diameter (in nm, see Table 3) is approximately *d*(300 K) = (3.43, 3.42, 3.41) and *d*(323 K) = (3.48, 3.47, 3.47). This corresponds to average volumes ranging from 20.98 to 21.90 nm^3^, respectively, representing an increase of about 4% due to the temperature rise. With the inclusion of [cho]­[gly] ion pairs, the bubble increases in volume. The water number density profiles in these systems (Figure 3b,c) show average diameters of approximately *d*(300 K) = 3.60 nm and d­(323 K) = 3.62 nm, with corresponding average volumes of 24.41 and 24.89 nm^3^, when only 20 ion pairs are present. This corresponds to volume increases of 16% and 14%, respectively, compared to the pure water bubble. When the number of ion pairs increases to 40, the average diameters become *d*(300 K) = 3.72 nm and *d*(323 K) = 3.84 nm, with volumes of 27.04 and 29.69 nm^3^, indicating increases of 29% and 36%. For the system composed of water and [emim]­[BF_4_] ion pairs, the same analysis is presented in Figure 4. The results show that, when the bubble contains 20 ion pairs, the average diameters are approximately *d*(300 K) = 3.58 nm and d­(323 K) = 3.64 nm, with corresponding volumes of 24.10 and 25.30 nm^3^, representing increases of 15% and 16% compared to the pure water bubble. These values are consistent with the behavior observed for [cho]­[gly]. For 40 pairs of [emim]­[BF_4_], we find an average diameter of *d*(300 K) = 3.77 nm and *d*(323 K) = 3.83 nm, with volumes of 27.98 and 29.43 nm^3^, corresponding to volume increases of 15% and 34%. These trends are in good agreement with previous studies reporting that ionic liquids, particularly those with hydrogen-bond-donating cations, expand water-rich domains and enhance local structuring. It is important to note that the increase in droplet volume cannot be explained solely by the larger molar volume of the ionic liquid components. The relative expansion observed (up to ∼36% for 40 ion pairs) exceeds the simple volumetric contribution expected from ion insertion and is accompanied by changes in the density symmetry and HB network cohesion. These features indicate that the volume increase originates from a cooperative structural reorganization of the water–ion interface rather than a purely steric effect. For instance, Dziubinska-Kühn et al.^8^ and Lundgren et al.^12^ reported increases in water domain organization and volume in the presence of protic ILs like [MIM]­[Cl], while Bernardes et al.^7^ showed that the solubility and dispersion of water in ILs depend strongly on the nature and concentration of the ions, often resulting in larger, more stable clusters—supporting the observed growth in droplet size as ion pair concentration increases.

**Table 3:** Average Droplet Diameters (*dx*, *dy*, *dz*), in nm, along the Three Cartesian Directions, Mean Diameter ⟨*d*⟩, and Corresponding Droplet Volume ⟨*V*⟩ (in nm^3^) Obtained from Molecular Dynamics Simulations at 300 and 323 K for Pure Water and Ionic Liquid–Containing Systems^a^

pure water	*dx*	*dy*	*dz*	⟨*d*⟩	⟨*V*⟩
300 K	3.43	3.42	3.41	3.42	20.98
323 K	3.48	3.47	3.47	3.47	21.90
System-A1					
300 K	3.59	3.60	3.60	3.60	24.41
323 K	3.56	3.66	3.65	3.62	24.89
System-A2					
300 K	3.59	3.58	3.59	3.58	24.10
323 K	3.64	3.64	3.64	3.64	25.30
System-B1					
300 K	3.80	3.76	3.60	3.72	27.04
323 K	3.84	3.85	3.83	3.84	29.69
System-B2					
300 K	3.77	3.77	3.76	3.77	27.98
323 K	3.84	3.83	3.83	3.83	29.43

a Systems A1 and B1 correspond to [cho]­[gly]-water droplets, while systems A2 and B2 correspond to [emim]­[BF_4_]-water droplets.

**Figure 4:**
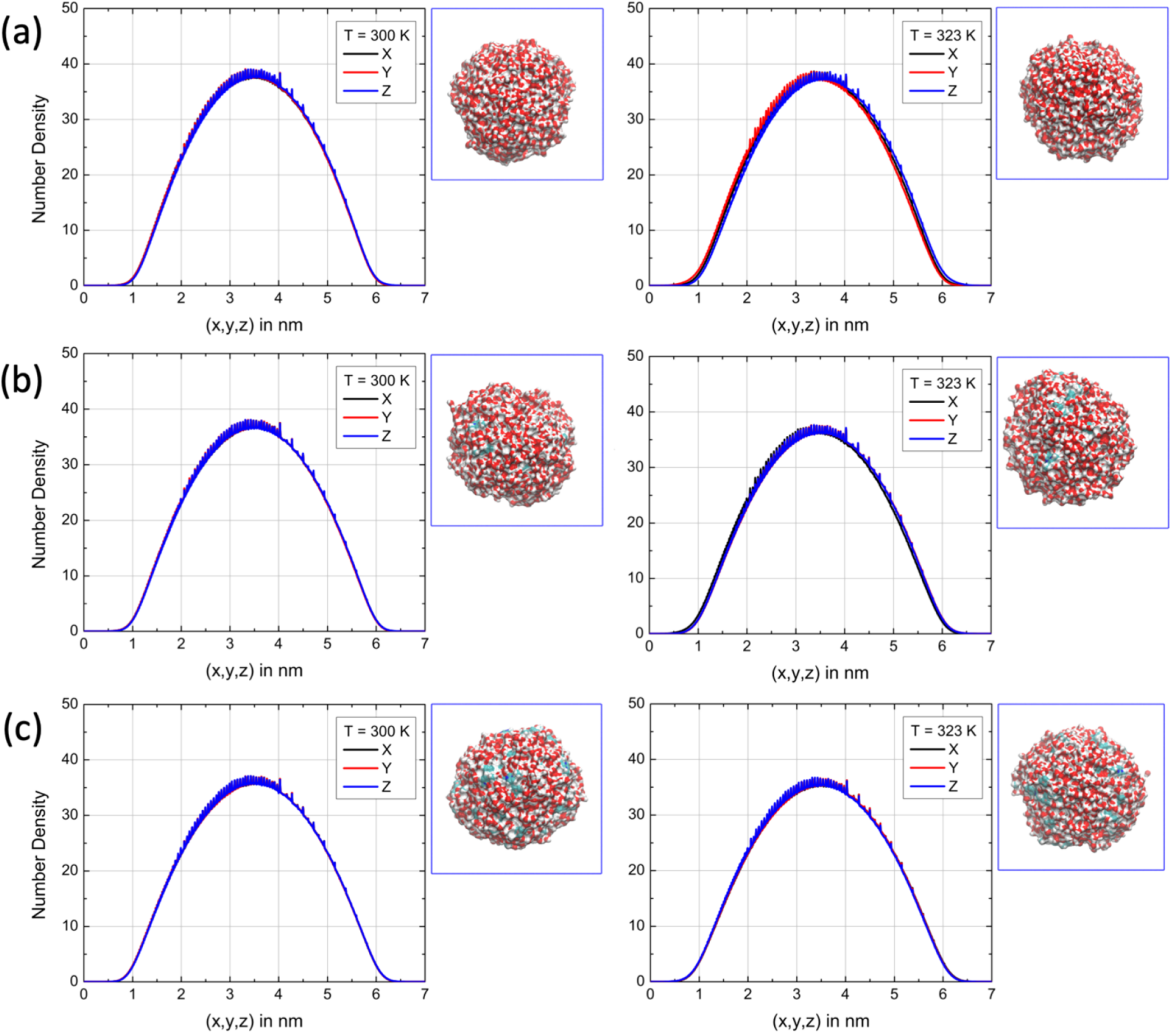
Final configuration from the simulation of (a) the pure water bubble; water bubble with (b) 20 pairs of [emim]­[BF_4_]; and (c) 40 pairs of [emim]­[BF_4_]. The number density projections along the three axes of the system are also shown for a simulation box with fixed edges of 7 nm. The *XY* plane is highlighted to illustrate the shape of the bubble.

The structural analysis of the water bubble, based on number density profiles, reveals that both temperature and the presence of ionic liquids significantly influence the system’s volume and spatial organization. The pure water bubble exhibits a symmetric and nearly spherical distribution, with a slight volume increase (∼4%) when the temperature rises from 300 to 323 K, consistent with expected thermal expansion. However, the introduction of ionic liquids, both protic ([cho]­[gly]) and aprotic ([emim]­[BF_4_]), induces a much more pronounced volume increase, proportional to the ionic pair concentration. The addition of 20 pairs leads to volume increases of up to ∼16%, while 40 pairs expand the volume by up to ∼36%, indicating that ions insert themselves into the interfacial region and reorganize the hydrogen-bond network, promoting droplet swelling. Among the studied ionic liquids, the [cho]­[gly] system exhibits slightly greater expansion than the [emim]­[BF_4_] system, particularly at 300 K. This is likely due to the stronger hydrogen-bonding capability of the choline cation and glycinate anion. Despite the volumetric expansion, the approximately spherical shape and symmetry of the density profiles are preserved in all cases, indicating that the structural integrity of the bubble is maintained even at high ion concentrations. These results demonstrate that ionic liquids can significantly reorganize the structure of confined water, with ion concentration having a greater impact than temperature. Such observations have important implications for controlling the morphology and stability of aqueous aggregates in nanoconfined environments.

### 3.3 Hydrogen Bond

To observe how ionic elements affect the structural consistency of the water bubble, we evaluated the average number of HBs and their dynamics (lifetime and free energy for HB rupture) according to the Luzar–Chandler and van der Spoel theory,^46–48^ considering a distance cutoff of *r* ≤ 0.35 nm and angle between donor-H-acceptor ≤30°. For the pure water bubble, our results show an average of 1.58 HBs per water molecule with a lifetime of approximately 2.4 ps and a free energy variation (Δ*G*) for HB rupture of around 6.7 kJ/mol at 300 K. These values decrease to 1.52 HBs per water molecule, with a lifetime of 1.8 ps (Δ*G* = 6.0 kJ/mol), representing a reduction of about 25% (11%) in HB lifetime (Δ*G*) at 323 K. Upon inclusion of 20 ion pairs of [cho]­[gly], the average number of HBs between water molecules remains close to 1.55 per molecule, with a lifetime of 2.7 ps (Δ*G* = 7.0 kJ/mol), very similar to the values observed for the pure water bubble at 300 K. However, at 323 K, the results (1.48 HBs per water molecule, 2.0 ps lifetime and Δ*G* = 6.3 kJ/mol) show that the presence of ions slightly extends the HB lifetime compared to the pure water model at this temperature, and the same applies to the Δ*G* values.

For the inclusion of 40 [cho]­[gly] ion pairs in the water bubble, the results show an average of 1.51 HBs per water molecule, with HB lifetimes of 3.0 ps and Δ*G* values around 7.3 kJ/mol, at 300 K. These results confirm that, even at the lowest simulated temperature, the presence of a protic ionic liquid diluted in the water bubble enhances the structural stability of the hydrogen-bond network among water molecules. Compared to the pure water bubble, we observe increases of approximately 28% in HB lifetime and 9% in HB rupture energy. At 323 K, the results further support our hypothesis and quantify the extent to which ionic interactions between water molecules and [cho] or [gly] help preserve the water–water network structure when solvating the ionic species inside the bubble. The results show approximately: 1.45 HBs per water molecule, 2.2 ps for HB lifetime, and Δ*G* = 6.5 kJ/mol, corresponding to increases of 25% in HB lifetime and 9% in Δ*G* compared to the values obtained for pure water at 323 K.

The analysis of hydrogen bonding dynamics within the nanoscopic water droplet revealed a clear influence of the ionic liquid [cho]­[gly] on the structural cohesion of the system. In the absence of ionic species, the number and lifetime of HBs naturally decrease with increasing temperature, consistent with the thermal disruption of the hydrogen-bond network. However, the inclusion of [cho]­[gly] leads to a significant stabilization of this network. The protic nature of both [cho]^+^ and [gly]^−^ enables them to participate in hydrogen bonding with water molecules, effectively reinforcing the overall structure. As a result, both the lifetime and the free energy required to break a hydrogen bond increase with the presence of these ions, even at elevated temperatures.

This behavior becomes more pronounced as the concentration of [cho]­[gly] increases, demonstrating a dose-dependent stabilization effect. At higher ion pair counts (40 ions), the HBs within the droplet not only persist longer but also exhibit greater resistance to thermal agitation, as evidenced by the elevated ΔG values. These findings highlight the protic ionic liquids in preserving hydrogen bond networks under thermal stress. Overall, [cho]­[gly] acts not merely as a solute but as an active component of the hydrogen-bond network, enhancing the internal structural integrity of the droplet and resisting temperature-induced disruption. For the droplet composed of water-[emim]­[BF_4_], the results obtained for the composition containing 20 pairs of [emim]­[BF_4_] show that the average number of HBs per water molecule is approximately 1.54 (1.48), with a lifetime close to 2.7 ps (2.0 ps) and Δ*G* around 7.0 kJ/mol (6.3 kJ/mol), for *T* = 300 K (*T* = 323 K). These results are very similar to those obtained for 20 pairs of [cho]­[gly]. For the composition with 40 pairs of [emim]­[BF_4_], the values are approximately 1.50 (1.44) HBs per water molecule, 3.0 ps (2.2 ps) for the lifetime of these interactions, and Δ*G* = 7.3 (6.5) kJ/mol for the HB breaking energy, for *T* = 300 K (*T* = 323 K). Table 4 show the comparison between results for water, water-[cho]­[gly] and water-[emim]­[BF_4_] at 300 and 323 K.

**Table 4:** Average Number of Hydrogen Bonds (# HBs) Per Water Molecule, HB Lifetime (in picoseconds), and the Free Energy Change (Δ*G* in kJ/mol) Required to Rupture a Hydrogen Bond^a^

system	*T* (K)	# HBs	HB lifetime	Δ*G*
Pure Water	300	1.58	2.4	6.7
Pure Water	323	1.52	1.8	6.0
System-A1	300	1.55	2.7	7.0
System-A1	323	1.48	2.0	6.3
System-B1	300	1.51	3.0	7.3
System-B1	323	1.45	2.2	6.5
System-A2	300	1.54	2.7	7.0
System-A2	323	1.48	2.0	6.3
System-B2	300	1.50	3.0	7.3
System-B2	323	1.44	2.2	6.5

a The data are presented for pure water bubbles and water bubbles containing different concentrations of the protic ionic liquid [cho]­[gly] and the aprotic ionic liquid [emim]­[BF_4_], at two temperatures: 300 and 323 K.

The analyses of HBs indicate that the presence of ionic liquids, plays a significant stabilizing role in the HBs network among water molecules within the droplet. This behavior can be attributed to the protic nature of these ions, which enables them to both donate and accept hydrogen bonds, fostering cooperative interactions with surrounding water molecules. As a result, even under increased thermal agitation (typically detrimental to HB structure in pure water) the network remains more cohesive in the presence of these ions. This effect is reflected in the higher average number of HBs, longer HB lifetimes, and increased free energy required for HB water–water rupture. Therefore, the ionic liquids act not merely as passive solutes, but as structural components that integrate into and reinforce the hydrogen-bonding network, enhancing the droplet’s thermal resilience and internal stability.

These results align well with previous studies showing that protic ionic liquids, due to their ability to donate and accept hydrogen bonds, significantly enhance the cohesion and structure of aqueous systems. For example, Matroodi et al.^5^ demonstrated that PILs like [MIM]­[Cl] increase the number and stability of water HBs by forming directional interactions, especially at low concentrations. Likewise, Dziubinska-Kühn et al.^8^ observed that water remains more tightly structured in IL-rich polar domains, where selective retention of water strengthens the local HB network. The increase in HB lifetime and rupture energy observed in our system is also consistent with the findings of Bernardes et al.,^7^ where simulations showed that certain ILs promote stronger water–anion coordination and reduce molecular mobility, enhancing the thermal robustness of the hydrogen-bond structure. Altogether, these comparisons confirm that IL-induced stabilization of water droplets, particularly through HB reinforcement, is a reproducible and robust phenomenon across various chemical environments and simulation conditions. Moreover, independent structural analyses of pure water droplets have shown that even in the absence of solutes, interfacial regions exhibit reduced tetrahedrality and lower local density compared to the droplet core, especially at elevated temperatures.^14^ This inherent surface fragility reinforces the view that additives capable of hydrogen bonding (such as PILs) play an essential role in preserving interfacial order. Altogether, these comparisons confirm that IL-induced stabilization of water droplets, particularly through HB reinforcement, is a reproducible and robust phenomenon across various chemical environments and simulation conditions.

Complementary dynamic information was obtained from the mean square displacement (MSD) analysis. For pure TIP3P water droplets, our calculated diffusion coefficient ( D ) of water molecules was estimated to be on the order of 3.3 × 10^–6^ cm^2^/s at 300 K, increasing to approximately 11.1 × 10^–6^ cm^2^/s at 323 K. In the presence of ionic liquids, both [cho]­[gly] and [emim]­[BF_4_] systems exhibited reduced water mobility, with D values around 2.7–2.9 × 10^–6^ cm^2^/s at 300 K and 8.7–11.0 × 10^–6^ cm^2^/s at 323 K, evidencing water–ions structural organization. The ionic pair showed lower diffusivities (<0.01 × 10^–6^ cm^2^/s), confirming the restricted translational motion of ions within the droplet. These results align with the increased hydrogen-bond lifetimes and higher interaction energies observed previously, reinforcing that the ionic liquids act as dynamic stabilizers that slow molecular diffusion while preserving droplet integrity. The comparative analysis of the diffusion coefficients further clarifies the connection between molecular mobility, droplet expansion, and hydrogen-bond stability. In the presence of protic ionic liquids ([cho]­[gly]), the translational mobility of water molecules is lower than in the aprotic system ([emim]­[BF_4_]), indicating stronger local structuring and more persistent hydrogen-bond networks. This reduced mobility correlates with both the larger volumetric expansion and the increased HB lifetime and rupture free energy (Δ*G*) observed for the [cho]­[gly] systems. The stronger water–ion hydrogen bonds and cooperative interactions with [cho]^+^ and [gly]^−^ create a more rigid interfacial framework, which resists molecular diffusion yet sustains cohesive expansion of the droplet. Thus, mobility, volume, and hydrogen-bond energetics are mutually consistent descriptors: protic ionic liquids reduce molecular diffusion while reinforcing the HB network and promoting droplet integrity under heating, whereas aprotic systems allow greater mobility and less cooperative cohesion.

The current simulations were conducted in the 300–323 K range to represent moderate thermal conditions relevant to both laboratory and atmospheric scenarios. Although the obtained trends are robust within this interval, extending these analyses to lower temperatures would require a systematic investigation beyond the present scope. Temperatures below 300 K may introduce additional structural regimes such as enhanced tetrahedrality, reduced mobility approaching the supercooled regime, and possibly altered droplet morphology. Therefore, a dedicated study—including extended simulation times, careful equilibration protocols, and potentially quantum corrections for HB energetics—would be necessary to ensure reliable results in that temperature domain. Such an analysis is planned as a natural continuation of this work to better connect molecular-level cohesion with atmospheric processes occurring under colder conditions.

### 3.4 Radial Distribution Functions

The analysis of the radial distribution functions, *g*(*r*), provides quantitative insight into the intermolecular organization and strength of local interactions within the ionic liquid–water systems. For the [cho]­[gly]-water droplets (Figure 5a), a prominent first peak appears at *r* ≈ 0.28 nm, characteristic of hydrogen bonding between the hydroxyl group of the cholinium cation, the carboxylate group of the glycinate anion, and surrounding water molecules. This peak remains well-defined even at 323 K, indicating that the hydrogen-bond network is robust and preserves its structural order under moderate heating. The slight decrease in peak intensity and the minor shift toward larger distances reflect only thermal expansion and increased translational mobility of water, without significant loss of interfacial cohesion. This stability arises from the protic nature of [cho]­[gly], which enables multiple cooperative hydrogen bonds with water molecules, producing a dense and structured interfacial region. In contrast, the *g*(*r*) profiles for systems containing [emim]­[BF_4_] (Figure 5b) display markedly different behavior. The first peak, centered at *r* ≈ 0.32 nm, is less intense and broader, indicating weaker and less directional interactions between the ions and water. This trend is consistent with the aprotic character of the imidazolium cation and the low hydrogen-bond acceptor capacity of the BF_4_^–^ anion, which promote more diffuse electrostatic coupling and a less rigid local structure. Increasing the temperature from 300 to 323 K further reduces the peak height. Consequently, water organization around [emim]­[BF_4_] becomes more thermally disordered, allowing enhanced molecular rearrangements and faster dynamics of the hydrated species. The direct comparison between protic ([cho]­[gly]) and aprotic ([emim]­[BF_4_]) systems highlights the key role of hydrogen bonding in maintaining water droplet structural stability. While [cho]­[gly] promotes cooperative organization between ions and water molecules [emim]­[BF_4_] is governed by long-range Coulombic and dispersion forces, resulting in a more diffuse and thermally labile interface.

**Figure 5:**
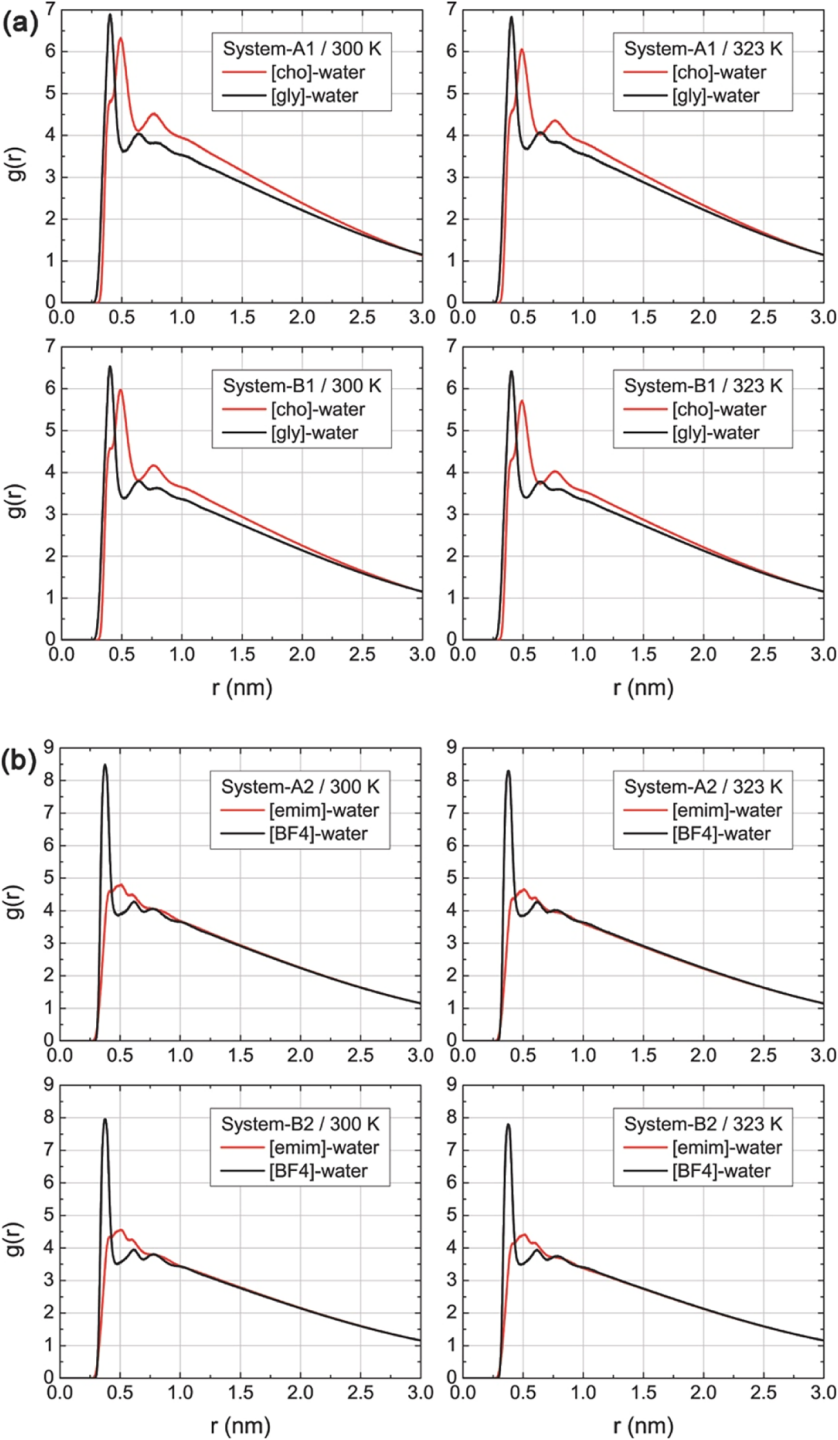
(a) Radial distribution functions, *g*(*r*), for ion–water pairs in droplets containing the protic ionic liquid [cho]­[gly] (systems A1 and B1) at 300 and 323 K. (b) Radial distribution functions for droplets containing the aprotic ionic liquid [emim]­[BF_4_] (systems A2 and B2) at 300 and 323 K.

### 3.5 Mechanistic Connections between Water-Based IL Systems and Atmospheric Aerosols

Aerosol literature shows that the water–solute interface controls hygroscopicity and morphological stability, as captured in κ-Köhler type parametrizations and mechanistic frameworks for SOA evolution.^22,25^ In our IL-in-water system, we observe preferential ion–water organization and cooperative reorganization of the HB network, which together modulate cohesion, local mobility, and thermal response. This mirrors the microinterfacial architecture of mixed atmospheric particles, where inorganic salts and polar organics form domains that regulate water uptake, effective surface tension, and interfacial diffusion.^22,24,25^ Across both contexts, the coupling between interfacial architecture and HB cooperativity governs condensed-phase trajectories and the hygroscopic response.

Atmospheric studies consistently depict water as a structural “glue”, organizing mixed phases and preventing phase separation under high humidity.^23,24^ In the present study, water preserves bubble integrity even under strong ionic competition imposed by the IL: the HB network self-adapts to counter perturbations, avoiding collapse of the confined aqueous cavity. Quantitatively, pure water bubbles expand modestly with temperature (∼4%), whereas IL addition drives much larger volume increases (up to 36% at high ion content) via ionic insertion and HB reorganization—yet density symmetry and near-spherical morphology are retained, evidencing water-mediated interfacial stabilization. The same principle explains the persistence of partially soluble atmospheric particles and their response to hygroscopic growth and CCN activation.^23,24^

For SOA, chemical nature (hydrophilic vs hydrophobic) governs water uptake, reactivity, and optical properties.^24,25^ An analogous duality emerges here: PILs behave like hydrophilic atmospheric constituents, reinforcing HBs (water ↔ ion and ion ↔ ion), stabilizing the bubble and enhancing cohesion; AILs resemble less-polar organics, with weaker HB interactions and lower water affinity, favoring interfacial reorganization. This composition-dependent HB modulation forms a bridge between molecular-scale IL–water interactions and mesoscale behavior of mixed atmospheric particles, implying a shared mechanism for phase stability, morphology, and optical response.

Temperature controls molecular mobility, diffusion, and internal organization, thereby modulating growth and activation in the atmosphere.^22,25^ Here, heating reduces *E*_C_/*E*_LJ_ and increases interfacial mobility, with partial HB network reorganization—yet the bubble maintains density symmetry and near-spherical shape, indicating that water-mediated cohesion persists under thermal stress. This maps onto atmospheric observations where diurnal and vertical thermal gradients accelerate molecular rearrangements, promote intraparticle diffusion, and can shift effective hygroscopicity, altering CCN activation thresholds.^22,25^

Large-scale climate models often condense hygroscopic behavior into simplified metrics, which do not fully capture molecular-scale interfacial physics.^22,25^ Our results provide mechanistic descriptors—HB numbers and lifetimes, rupture free-energy (Δ*G*), *E*_C_/*E*_LJ_ trends, and density symmetry—that can inform more physical parametrizations of water uptake and activation in complex particles. Incorporating the composition- and temperature-dependence of these descriptors should improve model skill under transient thermal regimes and multicomponent mixing, thereby reducing uncertainties in optics, radiative forcing, and removal.^22,25^

Therefore, interface, water-mediated stabilization, composition, and temperature converge to a unified picture in which water acts as a universal mediator of cohesion and interfacial organization. The quantitative descriptors derived from this study offer an operational bridge from IL–water nanoconfinement to aerosol microphysics, providing transferable metrics for parametrizing water uptake and activation in environmental models.^22–26^

## 4. Conclusion

The analysis of the structure and volume of water bubbles, both pure and containing protic ([cho]­[gly]) and aprotic ([emim]­[BF_4_]) ionic liquids, reveals that the presence of ions plays a decisive role in the spatial organization and volumetric behavior of the confined liquid phase. The bubble composed exclusively of water exhibits an almost spherical and symmetrical distribution, with a slight volumetric increase (∼4%) resulting from the temperature rise from 300 to 323 K, a phenomenon consistent with the expected thermal behavior of water due to natural volumetric expansion. When ionic liquids are introduced, a much more pronounced volumetric expansion is observed, dependent on ion concentration, with increases reaching up to 36% for 40 ion pairs compared to pure water. This growth suggests that the ionic molecules insert themselves in the interfacial regions of the bubble, promoting a reorganization of the hydrogen bond network of water and inducing swelling of the structure. Among the ionic liquids studied, the system with [cho]­[gly] shows greater volumetric expansion, likely due to its stronger ability to form hydrogen bonds, highlighting the specific chemical influence of the cations and anions on the structure of the aqueous phase. Although both ionic liquids differ slightly in ionic volume, the stronger stabilization observed for [cho]­[gly] cannot be attributed solely to molecular size. The enhanced cohesion arises primarily from the protic nature of this ionic liquid, whose functional groups promote additional directional hydrogen bonds with water molecules, resulting in longer HB lifetimes and higher rupture energies compared to the aprotic [emim]­[BF_4_].

Alongside the volumetric expansion, the dynamic analysis of hydrogen bonds within the bubbles confirms the stabilizing impact of the ionic liquids, especially [cho]­[gly], on the structural cohesion of the water network. In the pure water system, the temperature increase leads to a reduction in the average number of HBs per molecule, in their lifetime, and in the free energy required for their rupture, reflecting the typical thermal destabilization of the hydrogen bond network. Conversely, the addition of protic ionic liquids stabilizes these interactions, maintaining or even increasing the average number of HBs and prolonging their duration, as well as raising the energy barrier for bond breaking. This effect is dose-dependent, more pronounced at the higher concentration (40 ion pairs), indicating that the ionic species act as active structural elements capable of forming cooperative hydrogen bonds both with water molecules and among themselves. This reinforced network integration results in greater resistance of the bubble to thermal perturbation, maintaining its structural integrity even at temperatures higher than ambient.

Furthermore, the comparison between protic ([cho]­[gly]) and aprotic ([emim]­[BF_4_]) ionic liquids reveals striking similarities in the dynamic behavior of hydrogen bonds, with close values for average number of HBs, lifetimes, and free energies of rupture. However, [cho]­[gly] tends to show slightly greater stabilization, also due to the presence of a greater interaction energy together within longer lifetimes and higher free energies for rupture of the HBs, especially at lower temperatures. This subtlety can be attributed to the protic system’s enhanced ability to both donate and accept hydrogen bonds, thus expanding the cooperative network within the bubble. On the other hand, the system with [emim]­[BF_4_], although promoting similar effects (likely due to electrostatic interactions), shows less volumetric and stabilizing impact, suggesting a less ordered interaction with water (particularly hydrogen bonding), consistent with its aprotic nature.

In the context of bubble morphology, despite the considerable volumetric expansion caused by the presence of ionic liquids, the approximately spherical shape and the symmetry of the water molecule number density are preserved. This structural constancy indicates that, although the ions induce volumetric reorganizations and modifications in the hydrogen bond network, the overall integrity of the aggregate remains intact, which is crucial for potential applications relying on aqueous stability in confined environments. Thus, ionic liquids act not only as agents modifying the volume of a water droplet but also as elements strengthening the internal cohesion of the bubble, modulating its structural and even thermal stability (slightly above ambient temperature).

In summary, the correlation between volumetric properties and hydrogen bond dynamics highlights those ionic liquids (especially protic ones) function as structural stabilizers within aqueous bubbles, mitigating the effects of temperature increases on the HB network. This action results in greater resistance of the bubble to disintegration, significantly increasing its volume without compromising its shape and ensuring an internal robustness that can be exploited in systems where control of morphology and stability of aqueous nanoconfined aggregates is fundamental for applications. This understanding reinforces the active role of ionic liquids in interfacial chemistry and the engineering of nanoconfined systems.

Atmospheric parallels reinforce the generality of these mechanisms. In environmental particles, water governs hygroscopicity, particle growth, and cloud condensation nuclei activation, with interfacial organization determining optical properties, radiative forcing, and particle removal.^22–26^ The molecular descriptors quantified here—hydrogen-bond numbers and lifetimes, rupture free-energy, Coulombic and Lennard–Jones interaction trends, and density symmetry—thus emerge as transferable bridges between nanoconfined IL–water physics and aerosol microphysics, supporting more physically grounded parametrizations of water uptake and activation in atmospheric models.^22–26^

In summary, chemical composition (protic vs aprotic ILs) and temperature modulate interfacial cohesion through HB network adjustments and interaction energies. Nevertheless, water remains the key structuring element, conferring both thermal and compositional resilience. This conclusion resonates with evidence from atmospheric studies showing that water sustains morphological stability and functional properties even under variable thermal and compositional conditions.^23,24,26^ Collectively, these findings advance the understanding of complex aqueous interfaces and suggest multiscale modeling strategies in which molecular descriptors guide parametrizations capable of capturing nonlinear responses of mixed systems under realistic environmental variations.^22,25^
